# Histopathological and Immunohistochemical Evaluation of Canine Nerve Sheath Tumors and Proposal for an Updated Classification

**DOI:** 10.3390/vetsci9050204

**Published:** 2022-04-22

**Authors:** Kristina Tekavec, Tanja Švara, Tanja Knific, Mitja Gombač, Carlo Cantile

**Affiliations:** 1 Department of Veterinary Science, University of Pisa, 56124 Pisa, Italy; carlo.cantile@unipi.it; 2 Institute of Pathology, Wild Animals, Fish and Bees, Veterinary Faculty, University of Ljubljana, 1000 Ljubljana, Slovenia; tanja.svara@vf.uni-lj.si (T.Š.); mitja.gombac@vf.uni-lj.si (M.G.); 3 Institute of Food Safety, Feed and Environment, Veterinary Faculty, University of Ljubljana, 1000 Ljubljana, Slovenia; tanja.knific@vf.uni-lj.si

**Keywords:** dog, nerve sheath tumor, histopathology, immunohistochemistry, *Sox10*, claudin-1, GFAP, CNPase, Ki-67

## Abstract

Nerve sheath tumors are a group of tumors originating from Schwann cells, fibroblasts, and perineurial cells. In veterinary pathology, the terminology for nerve sheath tumors remains inconsistent, and many pathologists follow the human classification of such tumors in practice. Immunohistochemistry plays an important role in the diagnosis of nerve sheath tumors, but specific immunohistochemical and molecular biomarkers are lacking. In our study, we histopathologically reevaluated 79 canine nerve sheath tumors and assessed their reactivity for the immunohistochemical markers *Sox10*, claudin-1, GFAP, CNPase, and Ki-67. Based on the results, we classified the tumors according to the most recent human classification. Twelve cases were diagnosed as benign nerve sheath tumors, including six neurofibromas, three nerve sheath myxomas, two hybrid nerve sheath tumors (perineurioma/neurofibroma and perineurioma/schwannoma), and one schwannoma. Sixty-seven tumors were malignant nerve sheath tumors, including fifty-six conventional, four perineural, one epithelioid malignant nerve sheath tumor, and six malignant nerve sheath tumors with divergent differentiation. We believe that with the application of the proposed panel, an updated classification of canine nerve sheath tumors could largely follow the recent human WHO classification of tumors of the cranial and paraspinal nerves, but prospective studies would be needed to assess its prognostic value.

## 1. Introduction

Nerve sheath tumors (NSTs) are a group of tumors that arise from Schwann cells, perineurial cells, and epineurial or endoneurial fibroblasts. The four main subtypes of NSTs are, therefore, schwannoma (which consists exclusively of neoplastic Schwann cells), perineurioma (which consists of neoplastic perineurial cells), neurofibroma (which consists of a mixture of neoplastic Schwann cells, perineurial cells, and fibroblasts), and malignant NST (MNST), the first three being benign entities [1].

The current terminology associated with NSTs in veterinary texts is inconsistent and often confusing [1]. When properly classified, many of the NSTs observed in humans are also found in domestic animals, including their histological subtypes [2,3], leading many veterinary pathologists to follow the criteria and terminology of the human classification of peripheral NST in practice [1]. The latest, fifth edition and the sixth version of the human WHO Classification of Tumors of the Central Nervous System, published in 2021, classifies tumors of the cranial and paraspinal nerves into the following major subtypes: schwannoma, neurofibroma, perineurioma, hybrid nerve sheath tumor, malignant melanotic nerve sheath tumor, malignant peripheral nerve sheath tumor, and paraganglioma [4].

Based on the location of the tumors, taking into account their distance from the components of the nervous system, NSTs may belong to the root group, which involves nerves adjacent to the brainstem or spinal cord; the plexus group, which involves the brachial or lumbosacral plexus; or the peripheral group, which includes tumors arising distal to the brachial or lumbosacral plexus [5]. The most common locations of NSTs in dogs are the roots of the spinal nerves, especially at the level of the cervicothoracic spinal segment and in the brachial plexus and, occasionally, cranial nerves [1,6] and the skin [1,7]. Individual cases have also been reported in the liver [8], eye [9], eyelid [10], spleen [11], adrenal gland [12], diaphragm [13], lung [14], urinary bladder [15], tongue, and intestine [3].

NSTs may share considerable morphologic similarities with each other and also with other tumors not originating in the peripheral nervous system (PNS), which often makes diagnosis challenging. In addition, there are also some non-neoplastic proliferative lesions of the PNS that can mimic these tumors histologically [2]. In particular, in differentiating MNSTs from other soft tissue sarcomas, many authors agree that intrinsic nerve involvement contributes to the diagnosis of MNSTs in the absence of evidence of another specific line of differentiation. Another indicator is the presence of MNSTs in a previous benign NST (BNST). If the tumor has no relation to a nerve, the association between the morphologic features, immunohistochemistry (IHC), or ultrastructural features of the neoplastic cells is important for the diagnosis [16].

Although the identification of specific tumor cell types can be based to some extent on immunophenotyping and also electron microscopy, accurate data on the optimal panel of IHC markers in the diagnosis of these tumors are still lacking [1]. Many IHC markers expressed in NST have limited diagnostic sensitivity and specificity because their expression is often lost in MNST or expressed to varying degrees in other tumors. Therefore, the diagnosis of MNST is often based on the exclusion of other differential diagnoses using a comprehensive IHC panel [17].

The aim of our study is to histopathologically reevaluate 79 canine NSTs and to investigate the expression of Sry-related HMg-Box gene 10 (*Sox10*), claudin-1, glial fibrillary acidic protein (GFAP), 2′,3′-cyclic-nucleotide 3′-phosphodiesterase (CNPase), and proliferation marker Ki-67 to evaluate their correlation with histopathological criteria that may prove useful in the diagnosis of canine NSTs. In accordance with the latest human WHO classification, we will propose an update of the classification of NSTs in dogs.

## 2. Materials and Methods

### 2.1. Samples

In our study, we included 79 samples of canine tumors; 78 samples, collected between 2000 and 2022, were from the tissue archive of the Laboratory of Veterinary Neuropathology of the Department of Veterinary Science, University of Pisa (Italy), while one sample from 2021 was from the archive of Institute of Pathology, Wild Animals, Fish and Bees of the Veterinary Faculty, University of Ljubljana (Slovenia). The selected cases were previously diagnosed as NSTs based on the location and histopathological features of the tumors. The tumors were topographically classified based on their localization in the following groups: cranial nerve; cervical, cervicothoracic, thoracolumbar, or lumbosacral spinal cord segments; brachial or lumbosacral plexus; and appendicular nerve. The features and extent of the lesions were determined by the subjective evaluation of the pathologists involved in this study (K.T., T.Š., M.G. and C.C.).

### 2.2. Histopathology

All tumor samples were submitted to laboratories fixed in 10% buffered formalin and routinely embedded in paraffin. Formalin-fixed, paraffin-embedded (FFPE) tissue blocks were archived in the tissue archives. After retrieving the samples from the archives, we prepared 4 µm thick paraffin sections and stained them with hematoxylin and eosin (HE). We examined the HE slides under a light microscope and accurately and consistently evaluated the tissue and cellular characteristics of the tumors. Tissue criteria included evaluation of tumor shape, demarcation, encapsulation, growth type, cellularity, growth pattern, amount and type of stroma, extent of necrosis, extent of hemorrhage, blood and lymphatic vessel invasion, herniation into vessels, inflammatory infiltrates, hyalinization, and osseous and cartilaginous components. Cellular criteria included assessment of cell morphology, anisocytosis, anisokaryosis, cell margins, nuclear/cytoplasmic ratio, nuclear pleomorphism, nucleoli, number of mitoses per 10 high-power fields (HPF, 400× magnification—0.196 mm^2^), and presence of multinucleated cells. Details on the evaluation of the tissue and cellular criteria of tumors are provided in Table S1 (Supplementary Material).

We classified MNSTs into three histopathologic grades according to the soft tissue sarcoma (STS) classification system used in human pathology, which has also been used to grade cutaneous and subcutaneous STSs in dogs [18,19]. We used the grading system suggested for human NSTs by Rodriguez et al. [16], as shown in Table 1.

### 2.3. Immunohistochemistry

IHC staining for claudin-1, GFAP, and Ki-67 was performed using an automated IHC stainer, whereas IHC staining for *Sox10* and CNPase was performed manually. Paraffin sections of 4 µm thickness were prepared on positively charged slides. Sections were deparaffinized and rehydrated prior to performing the IHC staining protocols. Appropriate positive and negative controls from canine tissue were used in the staining procedures: lymph node as a positive control for Ki-67, brain for CNPase and GFAP, skin for claudin-1, and amelanotic melanoma for *Sox10*. Negative control included incubation with antibody diluent without the primary antibody. Sections were counterstained with Mayer’s hematoxylin and mounted. Details of the primary antibodies and immunohistochemical protocols used are given in Table 2.

To evaluate the expression of *Sox10*, GFAP, claudin-1, and CNPase, we used the four-point system of Adams et al., as follows [20,21]:

1. Strong (+++): dark staining that is clearly visible at low magnification and encompasses > 50% of cells.
2. Moderate (++): focal darkly stained areas encompassing <50% of cells or moderate staining of >50% of cells.
3. Weak (+): focal moderate staining in <50% of cells or pale staining in any proportion of cells that is not readily visible at low magnification.
4. Negative (−): none of the above.

The Ki-67 proliferation index was defined as the percentage of positive tumor cell nuclei to the 1000 nuclei counted in selected fields at 400× magnification. The proliferation index was determined in the areas subjectively identified as having the highest proportion of immunoreactive tumor cells.

### 2.4. Statistical Analysis

We performed the statistical analysis using the statistical software R, version 4.1.1 [22]. The difference in dog age, number of mitoses, and Ki-67 percentage between different groups according to tumor type and grading system was calculated using the Wilcoxon rank sum test and the Kruskal-Wallis rank sum test, respectively. The same tests were used to compare the proportion of Ki-67 by each marker separately. These tests were used because the variables were normally distributed. To compare the proportions between tumor type and grading system, we used Pearson’s chi-squared test or Fisher’s exact test if the assumptions for Pearson’s chi-squared test were violated. Since there were multiple comparisons with the same data set, we adjusted the *p*-values with a Benjamini–Hochberg correction. The correlations between the age of a dog, the number of mitoses, and the Ki-67 percentage were calculated with Spearman’s rank correlation coefficient, and the *p*-values were adjusted with Holm’s method. In all statistical tests, a *p*-value of less than 0.05 was considered statistically significant, while a *p*-value of less than 0.1 was interpreted as marginally statistically significant.

## 3. Results

### 3.1. Clinical Findings

Breed, sex, and age of the dogs, as well as main clinical signs and the site of the primary tumor, are shown in Table 3.

The majority of the dogs included in the study were a mixed breed (29/79, 36.7%). There were also 26 different purebred dog breeds represented, of which the German Shepherd was the most representative breed (9/79, 11.4%), followed by the Labrador Retriever (8/79, 10.1%). There were 59 male (74.7%) and 20 female (25.3%) dogs ranging in age from 1.5 to 13 years, with a median age of 8 years. The median age at diagnosis was 8 years (range 4–13) for MNSTs and 7.5 years (range 1.5–12) for BNSTs. Tumors were most frequently located at the roots of the spinal nerves (50/79, 63.3%), particularly at the level of the cervicothoracic spinal cord segment (group C, 25/50, 50.0%) and at the brachial plexus (group F, 16/79, 20.3%). A smaller number of tumors involved cranial nerves (group A, 6/79, 7.6%), appendicular nerves (group H, 5/79, 6.3%), and the lumbosacral plexus (group G, 2/79, 2.5%). All BNSTs originated from nerve roots from different spinal cord segments. Considering the available clinical information, clinical signs depended on the location of the tumor and usually included motor and sensory deficits due to compression or injury of nerves or the spinal cord. A cranial deficit and brainstem syndrome were, therefore, consistent with cranial nerve involvement; involvement of the brachial plexus or appendicular nerves was usually reflected in a lower motor neuron (LMN) syndrome, whereas extradural or intradural tumor extension and spinal cord compression often caused both LMN and upper motor neuron (UMN) symptoms. Metastases were reported in one case (no. 60), in which the brachial plexus tumor had metastasized to the lung and brain.

### 3.2. Histopathology and Immunohistochemistry

Based on their histopathologic and IHC features, 12 cases (15.2%) were diagnosed as BNSTs and 67 (84.8%) as MNSTs. Below, we describe the general histopathologic and IHC features of the different subtypes and their variants, whereas the exact diagnoses and expression of IHC markers for each case are listed in Table 4. A summary of the expression of IHC markers in the different subtypes and variants of NST is provided in Table 5, and a detailed IHC analysis of cases is provided in Table 6. IHC staining for Ki-67 was weak or without reaction and was considered unreliable for samples nos. 1, 2, 7, 19–29, 44, 45, 49, 51, 52, 57–60, and 75, submitted for histopathological examination between the years 2000 and 2008.

#### 3.2.1. Benign Nerve Sheath Tumors

Of 12 BNSTs, 6 cases were diagnosed as neurofibromas (nos. 17, 18, 31, 40, 42, 46), 1 was a schwannoma (no. 10), and 2 cases were hybrid NSTs—a perineurioma/neurofibroma (no. 23) and a perineurioma/schwannoma (no. 29). Three cases were designated as nerve sheath myxomas (nos. 12, 38, 52).

**Figure 1:**
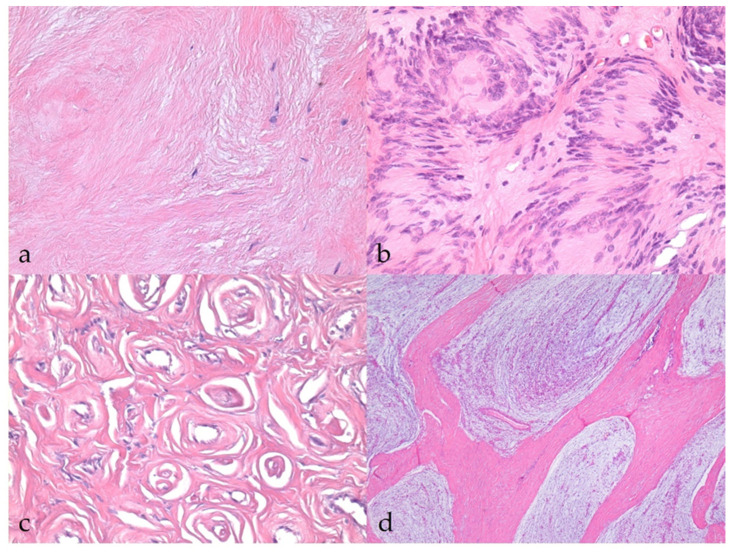
Histopathological characteristics of benign nerve sheath tumors (BNSTs). (**a**) Abundant collagenous stroma in the neurofibroma with the so-called ‘‘shredded carrot’’ appearance (case no. 17). HE, 400×. (**b**) Classic schwannoma with marked nuclear palisading (Verocay bodies) (case no. 10) HE, 400×. (**c**) Concentric arrangement of neoplastic perineurial cells, forming so-called pseudo-onion bulbs in the perineurioma regions of hybrid NST (case no. 23). HE, 400×. (**d**) Nerve sheath myxoma consisting of myxoid lobules separated by distinct collagenous septa (case no. 38). HE, 40×.

The majority of BNSTs presented as localized nodular masses, with the exception of one plexiform neurofibroma involving multiple nerves (no. 40). Five BNST specimens were incisional biopsies or incompletely excised, making it impossible to assess their demarcation, encapsulation, and growth type. The remaining BNSTs appeared as well-demarcated, encapsulated, or at least partially encapsulated masses confined to the epineurium. Occasionally, slightly infiltrative growth was observed longitudinally along the nerve. No atypical histologic patterns were noted in the BNSTs. No vascular invasion or herniation into vessels was observed in any of the BNSTs, whereas occasional hyalinization of blood vessels was noted. There were rare small necrotic areas and hemorrhages, the latter most likely artifacts of sampling. Multifocal inflammatory infiltrates were present, consisting mostly of lymphocytes and, occasionally, plasma cells. Tumor cells showed mild or no atypia, were mostly spindle- to stellate, sometimes elongated, with a small to moderate amount of eosinophilic cytoplasm, indistinct cell borders, and a single oval to round or wavy, hyperchromatic nucleus and only occasionally a small nucleolus. Zero mitoses were found in seven BNSTs, while the number of mitoses did not exceed 3/10 HPF in five other BNSTs. A moderate to abundant collagenous or myxoid stroma separated the tumor cells. The stromal collagen component in neurofibromas occasionally had the so-called “shredded carrot” appearance (no. 17) (Figure 1a). Foci of osseous metaplasia were noted in a hybrid perineurioma/neurofibroma (no. 23) and cartilaginous metaplasia in a hybrid perineurioma/schwannoma (no. 29) and one nerve sheath myxoma (no. 52), whereas both osseous and cartilaginous metaplasia were observed in another nerve sheath myxoma (no. 38). The metaplastic elements in BNSTs did not show atypia. Schwannoma (no. 10) exhibited a classic phenotype and was characterized by marked nuclear palisading (Verocay bodies) (Figure 1b). None of the specimens was diagnosed as perineurioma, but perineurioma regions were detected in both hybrid NSTs and were characterized by neoplastic perineurial cells arranged concentrically in multiple layers around centrally located axons, forming so-called pseudo-onion bulbs (Figure 1c). Three cases were diagnosed as nerve sheath myxoma due to the predominant myxoid stroma and stellate- and spindle-shaped tumor cells without atypia (nos. 12, 38, 52). Distinct collagenous septa separated the myxoid lobules and were particularly prominent in one case (no. 38, Figure 1d).

**Figure 2:**
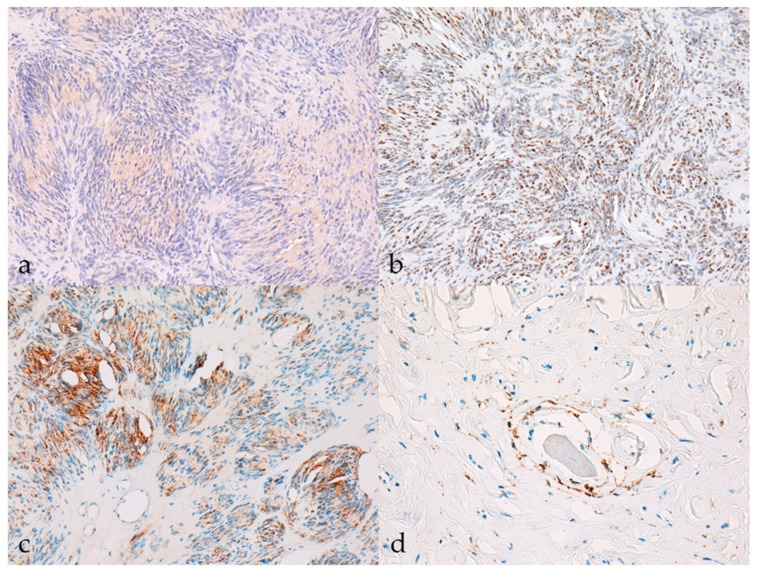
Immunohistochemical characteristics of benign nerve sheath tumors (BNSTs). (**a**) The cytoplasm of neoplastic cells in classical schwannoma shows mild immunoreactivity (+) for CNPase (case no. 10). CNPase, 200×. (**b**) Classical schwannoma showing diffuse strong nuclear immunoreactivity (+++) for *Sox10* (case no. 10). *Sox10*, 200×. (**c**) Multifocally (less than 50% of tumor), the cytoplasm of neoplastic cells in classic schwannoma moderately to strongly expresses GFAP (++) (case no. 10). GFAP, 200×. (**d**) Claudin-1 membranous immunoreactivity (++) of neoplastic perineurial cells in the perineurioma regions of hybrid NST (case no. 23). Claudin-1, 400×.

*Sox10*, claudin-1, and GFAP were differentially expressed in more than 90% of BNSTs, with moderate expression (++) being the most common, whereas CNPase was weakly expressed in only one BNST (classic schwannoma, no. 10, Figure 2a). The classic schwannoma was the only one that was completely negative for claudin-1 and showed strong reactivity for *Sox10* and moderate reactivity for GFAP (Figure 2b,c). Because of the lack of immunohistochemical reaction in the internal positive control (non-neoplastic Schwann cells), staining for *Sox10* and GFAP was considered unreliable in a hybrid perineurioma/neurofibroma (no. 23). Only one neurofibroma was negative for *Sox10*, but this was without an internal control to confirm the successful staining reaction. In hybrid perineurioma/schwannoma, *Sox10* and GFAP expression was restricted to the part of the schwannoma, whereas the perineurioma regions expressed claudin-1 moderately to strongly and were negative for *Sox10* and GFAP (Figure 2d). The Ki-67 proliferation index reached a maximum of 11.0% in BNSTs (mean 4.89 ± 4.06%).

#### 3.2.2. Malignant Nerve Sheath Tumors

Sixty-seven tumors (84.8%) were diagnosed as MNSTs. Fifty-six MNSTs (83.6%) were determined to be the conventional variant, six were with divergent differentiation (9.0%), four were perineural (6.0%), and one was an epithelioid MNST (1.5%). Histopathologically, 15 MNSTs (22.4%) were classified as grade I MNSTs, all of which were the conventional variant; 28 MNSTs (41.8%) were grade II, including 25 conventional, 2 perineural, and 1 epithelioid MNST; 24 MNSTs (35.8%) were grade III, including 16 conventional, 2 perineural, and all 6 MNSTs with divergent differentiation (Table 6).

**Figure 3:**
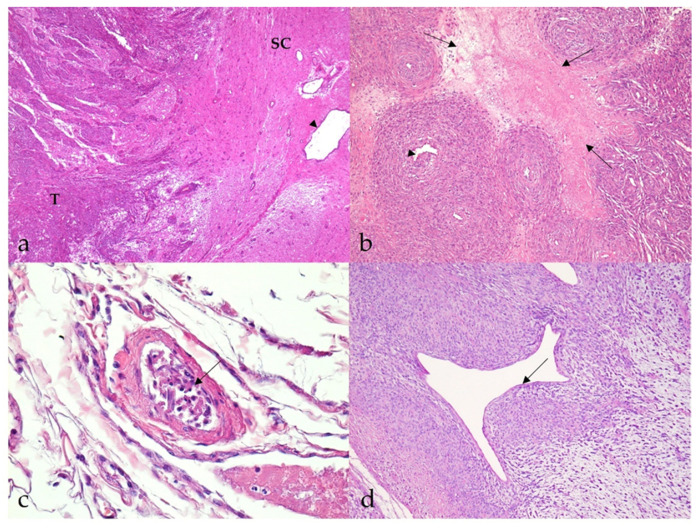
Histopathological characteristics of malignant nerve sheath tumors (MNSTs). (**a**) Marked infiltrative growth of high-cellular MNST into the spinal cord (case no. 51). T: tumor; SC: spinal cord; arrowhead: spinal cord canal. HE, 40×. (**b**) Well demarcated geographical necrosis (arrows). Slight intraluminal vascular herniation (arrowhead) is seen (case no. 69). HE, 100×. (**c**) Numerous spindloid cells in the lumen of a blood vessel, indicating blood vessel invasion (arrow) (case no. 60). HE, 400×. (**d**) Perivascular hypercellularity with distinct herniation of the tumor into the vessels (arrow) (case no. 65). HE, 100×.

**Figure 4:**
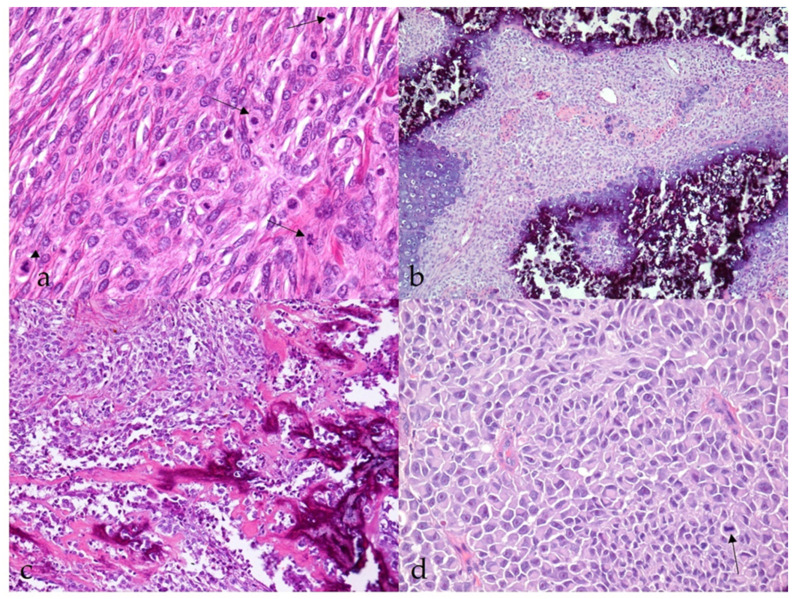
Histopathological characteristics of malignant nerve sheath tumors (MNSTs). (**a**) Brisk mitotic activity; few mitoses are indicated by an arrow. Occasionally, atypical mitoses are seen (arrowhead) (case no. 53). HE, 400×. (**b**) Chondrosarcomatous differentiation of MNST (case no. 78). HE, 100×. (**c**) Osteosarcomatous differentiation of MNST (case no. 39). HE, 100×. (**d**) Epithelioid MNST. The arrow indicates mitosis (case no. 79). HE, 400×.

Most MNSTs were poorly circumscribed, nonencapsulated tumors with infiltrative growth longitudinally along the nerve, occasionally into the spinal cord (Figure 3a), and, in some cases, through the epineurium into the surrounding tissue. They were usually highly cellular with a small to moderate amount of collagenous or myxoid stroma. In five conventional MNSTs (nos. 4, 6, 34, 61, 67), we suspected a transition from a neurofibroma because only portions of the tumor met the criteria for malignancy. Necrosis was common in MNSTs (44/65, 67.7%) and usually accounted for equal or less than 50% of the tumor (Figure 3b). The difference in the presence of necrosis between MNSTs and BNSTs was statistically significant (Fisher’s exact test, *p* = 0.0379). Blood vessel invasion was conspicuous in only one case of conventional grade III MNST (no. 60, Figure 3c), in which lung and brain metastases were already detected, whereas herniation of the tumor into the vessels was observed in 37.0% of MNSTs (24/65) (Figure 3d). The difference in herniation into vessels between MNSTs and BNSTs was statistically significant (Fisher’s Exact Test, *p* = 0.0355). No invasion into lymphatic vessels was detected. Intra- and/or peritumoral inflammatory infiltrates of varying sizes were common (44/65, 67.7%), with lymphocytes predominating, but the test didn’t show a statistically significant difference between MNSTs and BNSTs (Fisher’s exact test, *p* = 0.4866). Conventional and perineural MNSTs and spindle-cell components of MNSTs with divergent differentiation were usually characterized by fascicles, interlacing bundles, and concentric whorls of tumor cells. Tumor cells were spindle-shaped, fusiform, and occasionally oval, with higher-grade tumors showing greater cellular pleomorphism and atypia. The number of mitoses varied and ranged from a very low number such as 1 mitosis/10 HPF in low-grade MNSTs to an extremely high number of 105 mitoses/10 HPF in high-grade MNSTs, whereas the mean mitotic count was 17 ± 21 mitoses/10 HPF (Figure 4a). The difference in mitotic count between MNSTs and BNSTs was statistically significant (Wilcoxon rank sum test, *p* < 0.0001). All MNSTs with divergent differentiation contained osseous tissue and, with one exception (no. 66), also cartilaginous tissue. The osseous and, in two cases, cartilaginous components in three MNSTs with divergent differentiation (nos. 39, 66, 78) showed severe atypia, indicating osteosarcomatous and chondrosarcomatous differentiation (Figure 4b,c). A distinct epithelioid variant of MNST (no. 79) consisted of round and oval tumor cells with slightly eccentrically located round, oval, or bean-shaped nuclei in numerous cells (Figure 4d).

**Figure 5:**
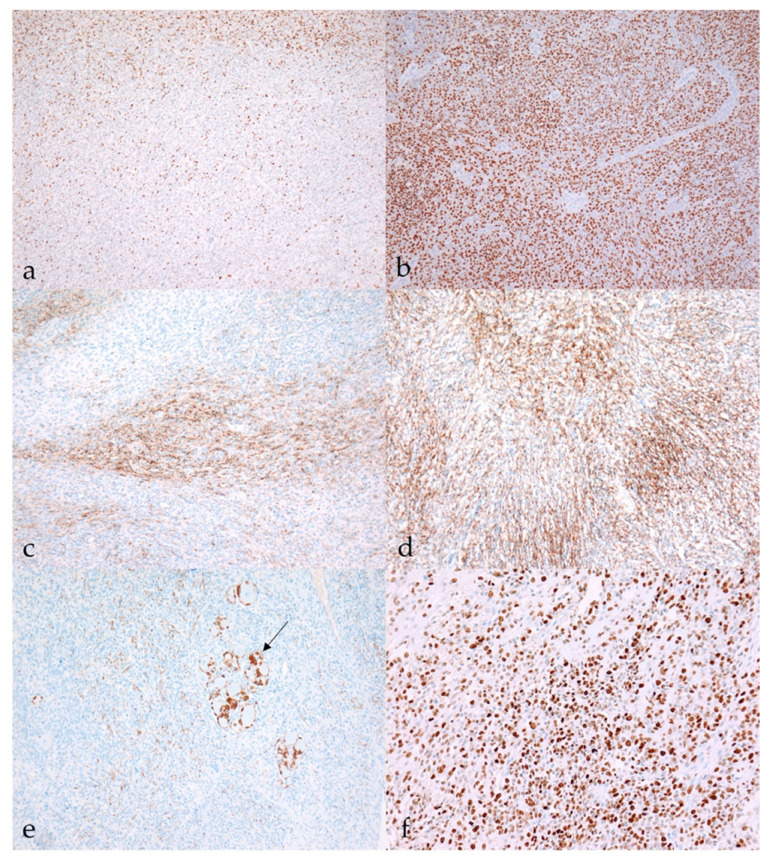
Immunohistochemical characteristics of malignant nerve sheath tumors (MNSTs). (**a**) Moderate nuclear immunoreactivity (++) for *Sox10* in conventional MNST (case no. 20). *Sox10*, 100×. (**b**) Strong diffuse nuclear immunoreactivity (+++) for *Sox10* in epithelioid MNST (case no. 79). *Sox10*, 100×. (**c**) Patchy membranous claudin-1 expression (++) in conventional MNST (case no. 69). Claudin-1, 100. (**d**) Strong membranous immunoreactivity (+++) for claudin-1 in MNST with perineurial differentiation (case no. 58). Claudin-1, 100×). (**e**) Moderate cytoplasmic immunoreactivity for GFAP (++) in conventional MNST. Arrow shows GFAP-positive non-neoplastic Schwann cells condensed around neuronal bodies (case no. 21). GFAP, 100×. (**f**) MNST with divergent differentiation had a Ki-67 proliferation index of 70.1% (case no. 39). Ki-67, 200×.

**Figure 6:**
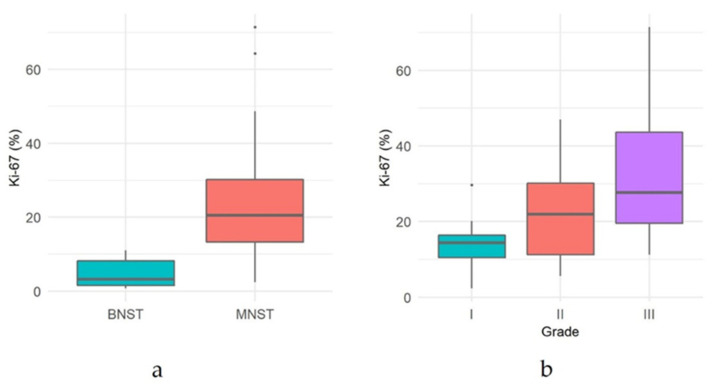
(**a**) Comparison of proliferation index Ki-67 between malignant nerve sheath tumors (MNSTs) and benign nerve sheath tumors (BNSTs). (**b**) Comparison of proliferation index Ki-67 between different grades of malignant nerve sheath tumors.

**Figure 7:**
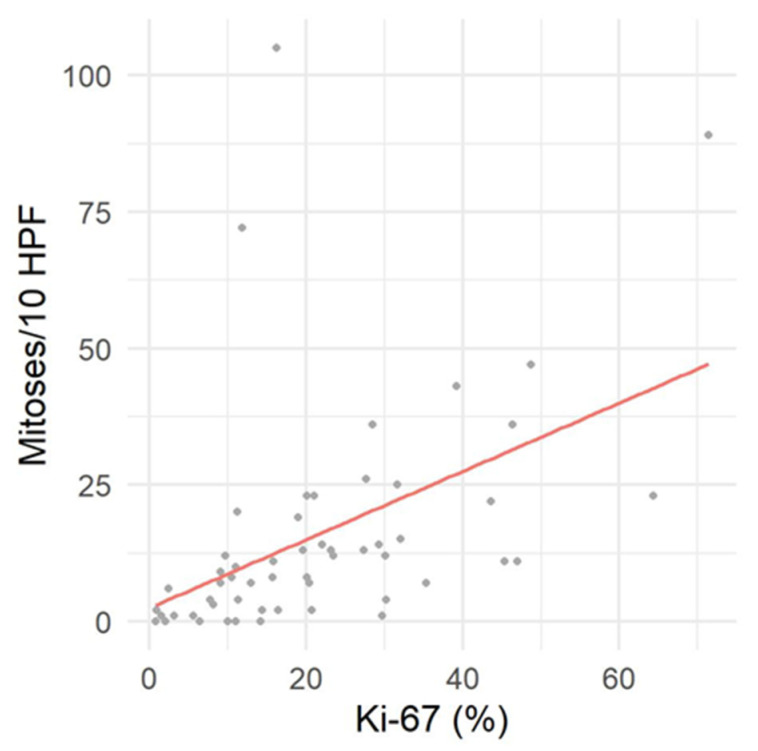
Correlation between proliferation index Ki-67 and mitotic count per 10 high power fields (HPF).

Immunoreactivity for *Sox10* was detected in 66.7% of MNSTs, with approximately half of the cases mildly expressing *Sox10* and the other half showing moderate expression (Figure 5a). Strong expression of *Sox10* was detected in only two MNSTs—an epithelioid MNST that was negative for other markers (Figure 5b) and a conventional MNST that also weakly expressed claudin-1. Claudin-1 was expressed in 70.8% of MNSTs, with moderate to strong immunoreactivity (Figure 5c). Of seven MNSTs that were strongly positive for claudin-1, four expressed only this marker and were consistently classified as MNSTs with perineural differentiation (Figure 5d). GFAP was mostly weakly expressed in 32.8% of MNSTs (Figure 5e), whereas all MNSTs were negative for CNPase. Twelve MNSTs were negative for all markers. Of these 12, 1 was classified as grade I, 4 as grade II, and 7 as grade III. Ten immunonegative MNSTs were conventional (nos. 4, 7, 9, 16, 19, 25, 56, 57, 60, 73), and two were MNSTs with divergent differentiation (nos. 1, 44) but with metaplastic osseous and cartilaginous tissue that did not show atypia. The highest proliferation index Ki-67 of one MNST with divergent differentiation was 71.4% (Figure 5f), whereas the mean Ki-67 proliferation index in MNSTs was 24.41 ± 15.07%. The difference in Ki-67 between MNSTs and BNSTs was statistically significant (Wilcoxon rank sum test, *p* = 0.0001), as visible in Figure 6a. Figure 6b shows the difference in the Ki-67 proliferation index between the different MNST grades, which was marginally statistically significant (Kruskal–Wallis rank sum test, *p* = 0.0907). Spearman’s rank correlation coefficient showed a high correlation between Ki-67 and the number of mitoses/10 HPF (*p* < 0.0001), as shown in Figure 7, where a higher number of mitoses was associated with a higher Ki-67 proliferation index.

## 4. Discussion

The diagnosis of NSTs is often challenging for pathologists, particularly in distinguishing MNSTs from STSs or, when the tumor lacks obvious malignancy criteria, from BNSTs [2,16]. While the prognosis for BNSTs is generally excellent, MNSTs tend to have a poor prognosis [23], making it all the more necessary to establish histopathological criteria that could help predict clinical outcomes and possible treatment plans. In our study, we reexamined 79 tumors in dogs, previously diagnosed as NSTs. Based on the localization, which confirmed the origin of the tumors from the nerve sheath, we were able to test their immunoreactivity to *Sox10*, claudin-1, GFAP, CNPase, and Ki-67 and evaluate the potential role of these IHC markers in the diagnosis of NSTs in dogs.

In the samples included in our study, we recognized histopathologic and IHC features of canine NSTs that resembled their human counterparts. However, in human patients, many of the specific NSTs are associated with specific germline or somatic mutations and occur as part of familial tumor syndromes—neurofibromatosis type 1 (NF1), neurofibromatosis type 2 (NF2), and schwannomatosis [24]—while in veterinary medicine, data on genetic disorders associated with the occurrence of neoplasms are still sparse.

A small number of tumors were benign and consistent with the diagnoses of schwannoma, neurofibroma, and hybrid BNST, the latter consisting of perineurioma in combination with schwannoma or neurofibroma areas. Although our study included perineurioma only as the component of hybrid BNSTs, the literature in veterinary medicine describes few individual cases of intraneural perineurioma in dogs [25,26,27]. The abundant myxoid stroma in three BNSTs led us to the diagnosis of nerve sheath myxoma, although the diagnosis may not be entirely appropriate. There are a number of reports of this entity in humans, where it is characterized as a cutaneous neoplasm of nerve sheath origin and is classified as a subtype of soft tissue tumor in the human WHO classification [28]. The authors emphasized diffuse positivity for S100, confirming the origin of the tumor from Schwann cells [29,30]. According to that, one would expect diffuse *Sox10* expression in this variant of BNST, but our cases showed only mild to moderate positivity for *Sox10* and GFAP and, in addition to that, also mild to moderate immunopositivity for claudin-1. Although the phenotype fits the description of a nerve sheath myxoma, this variant in the dog can be considered a benign myxoid NST for the time being [3]. Further immunohistochemical and ultrastructural studies of this histopathologic variant in dogs would be needed to define it more precisely.

The MNSTs that accounted for the majority of NST cases submitted to our laboratories between 2000 and 2022 were conventional, divergent, perineural, and epithelioid histopathologic variants. They were divided into low-grade (grade I), intermediate-grade (grade II), and high-grade (grade III) MNSTs according to the criteria of the STS grading system. Unfortunately, we do not have data on the disease course and survival of the patients whose tumors were included in our study. This would be necessary to evaluate the applicability of the grading system and the significance of the classification of MNSTs into different histopathological variants. Future prospective studies will be needed to investigate them.

We used a high Ki-67 proliferation index as an indicator of malignancy because it has been described as an important prognostic indicator in humans, with an elevated index indicating a worse prognosis [17,31,32]. However, caution should be taken not to diagnose the tumor as malignant too quickly, as Ki-67 values of MNSTs and cellular schwannomas may overlap. In their study, Pekmezci et al. found a Ki-67 index ranging from 1% in MNSTs and up to 36% in human cellular schwannomas, while no metastatic potential and no disease-related deaths demonstrated the benign nature of the latter [33]. Unfortunately, we could not calculate the Ki-67 index in all tumors because immunoreactivity for Ki-67 proved unreliable in 24 of the samples submitted between 2000 and 2008. We believe that the unsuccessful staining may be the result of poor antigen preservation in the old archival blocks, as Ki-67 has been shown to be a highly problematic nuclear marker characterized by marked antigen decay, leading to a reduction of immunosignal intensity in archival tissues [34]. Grillo et al., who studied antigen preservation in FFPE tissues, presented two strategies that proved useful for antigen recovery: deep sections and prolonged heat pretreatment [34]. We attempted to recover antigen through deeper sections, which proved useful in some of our cases but not in these 24. Since our antigen retrieval already took 60 min, we avoided a longer retrieval because it would most likely destroy the sample.

The most common variant in our case series was conventional MNST, which accounted for 83.6% of the included tumors. Of the conventional MNSTs, 26.8% were grade I, 44.6% were grade II, and 28.6% were grade III. One conventional MNST of grade III was metastatic, while others were not found to have metastases, but we could not exclude them because the follow-up for these dogs was unfortunately unknown. With the increasing grade of conventional MNSTs, we recorded a slight decrease in the expression of IHC markers *Sox10*, claudin-1, and GFAP, which could be the result of poorer differentiation of tumor cells in higher-grade tumors. The proliferation index Ki-67 increases with grade, which is consistent with the prediction of the more malignant nature of higher-grade tumors, but the difference between the different histological grades was only marginally statistically significant. On the other hand, our results showed a statistically significant difference between MNSTs and BNSTs.

MNSTs with divergent differentiation accounted for 9.0% of our MNST and were all classified as grade III MNSTs based on their histopathologic features. MNSTs with divergent differentiation are associated with poor prognosis [35]. In humans, they often occur in association with NF1 and correlate prognostically with conventional high-grade MNSTs. It may involve areas of neoplastic cartilage, bone, skeletal muscle, smooth muscle, or angiosarcoma-like areas. MNSTs with rhabdomyosarcomatous differentiation are also known as malignant Triton tumors. In addition to mesenchymal tissue, the tumors may also contain glandular or neuroendocrine epithelium and, rarely, squamous epithelium [24]. The exact reason for the divergent differentiation of MNSTs is unclear, but a reasonable explanation may lie in the pluripotency of the cells of the neural crest. These are highly migratory cells that give rise to various derivatives, including neurons and glia of the sensory, sympathetic, and enteric nervous systems, melanocytes, and cartilaginous, bony, and connective tissues of the head and neck [23,36]. Divergent differentiation has been described previously in canine NSTs. Anderson et al. were among the first to describe a case of MNST with chondro-osseous differentiation originating from the diaphragm of a 1-year-old dog [13]. Only a few years later, Patnaik et al. described a case of MNST in a dog with osteosarcomatous and glandular differentiation [35], and Kim et al. reported MNST in a dog with osteosarcomatous, rhabdomyosarcomatous, and myxomatous differentiation [37]. The study by Chijiwa et al. included two cases of MNST with cartilaginous and osseous metaplasia [23]. However, MNST with divergent differentiation might represent a histopathological pattern rather than a variant. In contrast to variants, which have potential clinical utility, the different histopathological patterns, such as malignant Triton tumor or glandular MNST, both of which belong to the group of MNSTs with divergent differentiation, usually have no clear clinicopathological significance [24].

Four MNSTs (6.0%) were strongly immunoreactive for claudin-1 and negative for *Sox10* and GFAP and were accordingly classified as MNSTs with perineurial differentiation or malignant perineurioma. MNSTs with perineurial differentiation appear to be less aggressive than conventional NSTs in humans, although they have the potential to metastasize [24]. Malignant perineuriomas have not yet been described in dogs. In the study by Jakab et al., who examined claudin-1 expression in canine NSTs, no claudin-1-positive and S100-negative reaction was detected within a single malignant tumor—this result would support a diagnosis of malignant perineurioma [38]. In their publication, they mention the study by Chijiwa et al., in which they described three S100-negative MNSTs in dogs, which could be MNSTs with perineurial differentiation. However, this has not been proven [23,38].

Finally, one MNST (1.5%) in our case series was compatible with a diagnosis of epithelioid MNST based on tumor cell morphology and diffuse expression of *Sox10*, which strongly supported such a diagnosis. Epithelioid MNST is a variant that can arise from the malignant transformation of a schwannoma. In humans, it shows no association with NF1, and the risk of recurrence, metastasis, and disease-related death appears to be lower compared to conventional MNSTs [24]. In dogs, few cases of epithelioid MNSTs have been described in the literature [39,40]. In one case, metastases were found in the liver, kidneys, lungs, and lymph nodes [39].

A change in nomenclature in human classification represents the renaming of the former melanotic schwannoma to malignant melanotic NST as it has been recognized as a very characteristic and often aggressive tumor type with a unique genetic basis [4]. Since *Sox10* also labels melanocytes, the differentiation of melanotic NSTs from melanocytic tumors requires the use of *Sox10* in conjunction with laminin or collagen IV [2]. Our study did not include a melanotic tumor. However, six cases of invasive melanotic NSTs in dogs consisting of neoplastic proliferation of pigmented Schwann cells and exhibiting numerous criteria of malignancy have been reported [41,42]. We believe that such findings are more consistent with a diagnosis of malignant melanotic NST than melanotic schwannoma as the latter can be quickly misinterpreted as BNST.

In the most recent human WHO classification, paraganglioma was included in the group of nerve tumors because it involves specialized neuroendocrine cells of the sympathetic and parasympathetic nervous systems [4]. In humans, paragangliomas arising in the head and neck are usually nonproducing, whereas those arising in the thoracic and abdominal cavities are more likely to produce catecholamines. In dogs, the classification of paragangliomas is not well defined because the information is lacking and mostly limited to case reports. Described cases in dogs often originate in the aorta or carotid body [43,44]. In our opinion, the inclusion of paraganglioma in the group of NSTs in dogs could be considered, but a deeper insight into this particular entity would be needed.

Based on our results, we consider *Sox10*, claudin-1, GFAP, and Ki-67 useful in the diagnosis of NST, whereas CNPase was negative in almost all cases, except for classical schwannoma, which was only mildly positive.

*Sox10* is a transcription factor that is crucial for the specification, maturation, and maintenance of Schwann cells and melanocytes [45,46]. It is not completely specific as it has also been expressed in myoepithelial cells of mammary tissue and in myoepithelial and acinar cells of salivary gland tissue [47]. In our study, we detected the expression of *Sox10* in 68.4% of all NSTs. It was expressed in 90.9% of BNSTs and 66.7% of MNSTs. In BNSTs, strong expression was detected only in classical schwannoma, consistent with the histopathological diagnosis based on the typical phenotype. Complete negativity for *Sox10* was observed in one neurofibroma and in the perineurioma portion of a hybrid perineurioma–schwannoma. In perineurioma, the negative result is consistent with a uniform population of neoplastic perineurial cells, recently shown to be non-neural crest-derived and possibly neuroectoderm-derived [48]. In contrast, one would expect variable *Sox10* reactivity in neurofibroma. Since there were no obvious nerve remnants that could have served as an internal positive control, we cannot be certain that the staining procedure was successful. As for MNST, 66.7% of tumors in our study expressed *Sox10* to varying degrees, which is consistent with the human MNST staining results of other authors, such as Kang et al. (67%) [21], Ersen et al. (54%) [49] and Nonaka et al. (49%) [46], and slightly higher than the results of Karamchandani et al. (27%) [47]. *Sox10* is less expressed in MNSTs than in BNSTs, and expression also decreases in higher-grade MNSTs compared with low-grade tumors. This trend most likely reflects the lower degree of Schwann cell differentiation in more malignant tumors. In humans, the difference in expression is also associated with NF1, whereas there are no known familial genetic mutations for NSTs in dogs [49].

Claudin-1 belongs to the claudin family, a group of at least 24 different integral membrane proteins that play an important role in tight junctions of epithelial and endothelial cells [50]. Claudin-1 is known to be widely expressed in epithelia in general and has also been found in normal and neoplastic perineurial cells, which are known to form tight junctions according to their role in the blood–nerve barrier [48,51]. Our results show that claudin-1 is expressed in 74.7% of NSTs, with immunoreactivity detected in 91.7% of BNSTs and 71.6% of MNSTs. Whereas schwannomas and epithelioid MNSTs that were diffusely *Sox10*-positive and apparently derived from Schwann cells were completely negative for claudin-1, perineuriomas and perineurial MNSTs showed moderate to strong claudin-1 positivity. On the basis of our results, we agree with previous studies in human and veterinary medicine that have shown that claudin-1 in combination with other antibodies could serve as a useful marker to distinguish NSTs from other spindle cell tumors and, moreover, to subclassify NSTs [25,38,51]. Nevertheless, Jakab et al. (2012) also reported claudin-1 positivity in canine hemangiopericytomas and myopericytomas, highlighting the importance of using a combined IHC panel to rule out differential diagnoses [38].

GFAP, also known as plaque protein or GFA protein, belongs to the cytoskeletal protein family and is the major intermediate filament (IF) in mature astrocytes. It plays an important role in regulating astrocyte motility and shape by providing structural stability to extensions of astrocytic processes [52]. It also acts as a support for neighboring neurons and the blood–brain barrier. In addition, it is found in nonmyelinating Schwann cells in the peripheral nervous system and in enteric glial cells [53]. It is not present in neoplasms of mesenchymal origin [54]. In our study, GFAP expression was detected in 43% of NSTs. It was mildly to moderately expressed in all BNSTs (100%) and mostly mildly expressed in 32.8% of MNSTs. According to the literature in human and veterinary medicine, the expression of GFAP varies in NSTs [54,55,56,57]. Gray et al. considered the reason for variable expression to be differences in methodology and in the specificity and sensitivity of the different antibodies used. Additionally, sequential changes in the expression of intermediate filaments (IF) during tumorigenesis could explain some apparent variations in the IF complement of NSTs and other neoplasms [55]. Stanton et al. suggested an influence of tumor location on GFAP immunoreactivity [58]. NSTs expressing GFAP may arise from or involve nerves with more nonmyelinated Schwann cells. The results of the Kawahara et al. study also suggest that GFAP is more frequently expressed by tumors located deeper in the spinal canal, mediastinum, and other organs where nonmyelinated fibers are present, although this cannot be considered certain [56]. Although GFAP is expressed in only a small percentage of MNSTs, we believe that it may still be useful in some cases to distinguish NSTs arising outside the CNS from other spindle cell tumors.

CNPase is a myelin-associated enzyme localized almost exclusively in the two cell types responsible for myelin sheath formation—oligodendrocytes and Schwann cells, respectively [59,60]. Although it is expressed in Schwann cells, there are limited data on its potential usefulness in NSTs. Nielsen et al. have shown that it is a sensitive marker for bovine NSTs [61], whereas, to our knowledge, it has not yet been tested in NSTs of other animal species or humans. We have detected mild CNPase immunoreactivity in only one canine NST—a classical schwannoma. Based on our results, we do not consider CNPase useful in the diagnosis of canine NSTs.

## 5. Conclusions

In conclusion, considering our findings and incorporating data from the literature, we believe that an updated classification of NST in dogs could largely follow the recent human WHO classification of tumors of the cranial and paraspinal nerves. We have identified *Sox10*, claudin-1, GFAP, and Ki-67 as useful IHC markers, whereas CNPase has no value for the diagnosis and classification of NSTs in dogs, according to the results of our study.

However, there is still a need for a larger prospective study to investigate the histopathological patterns and expression of various IHC markers in relation to disease progression and survival to determine their prognostic utility. In addition, our study examined only the sensitivity, not the specificity, of IHC markers. Additional IHC studies that include other tumor types would be needed, particularly those that are most common differential diagnoses for NSTs. Because NSTs in humans are often associated with genetic mutations, more detailed insight into genetics is needed to identify potential genetic alterations associated with NSTs in dogs.

## Figures and Tables

**Table 1:** Grading system for STS modified for MNST.

*Differentiation Score*	
1	Well-differentiated MNSTs arising in transition from neurofibroma
2	Conventional, monomorphous spindle cell MNSTs
3	Highly pleomorphic MNSTs, as well as MNSTs with divergent differentiation
*Mitotic count*	
1	0–9 mitoses/10 HPF
2	10–19 mitoses/10 HPF
3	>19 mitoses/10 HPF
*Tumor necrosis*	
0	No necrosis
1	≤50% necrosis
2	>50% necrosis
Histological grade *	
I	≤3
II	4–5
III	≥6

STS: soft tissue sarcoma. MNST: malignant nerve sheath tumor. HPF: high-power fields. * Histological grade corresponds to the sum of all three parameters assessed—differentiation score, mitotic count, and tumor necrosis.

**Table 2:** Details of the primary antibodies and immunohistochemical protocols.

Primary Antibody, Clone, and Catalogue Number	Manufacturer	Antigen Retrieval	Antibody Dilution	Time and Temperature of Incubation of the Primary Antibody	Detection System	IHC Automated Stainer
Ki-67, MIB-1, (M7240)	Dako, Denmark	CC1, pH 8.5, 60 min, 25 °C	1/50	32 min, 37 °C	UltraView Universal DAB Detection Kit (Ventana Medical Systems Inc., Tucson, AZ, USA)	Ventana Benchmark XT (USA)
CNPase, 11-5B, (ab6319)	Abcam, UK	Citrate buffer, pH 6.0, MW (1100 W), 20 min	1/750	60 min, 23 °C	DAKO REAL^TM^ EnVision Detection System Peroxidase/DAB+, Rabbit/Mouse (Dako, Denmark)	/
Claudin-1, (ab15098)	Abcam, UK	ULTRA CC1, pH 8.45–8.65, 56 min, 25 °C	1/50	20 min, 37 °C	OptiView DAB Detection Kit (Ventana Medical Systems Inc., Tucson, AZ, USA)	Ventana Benchmark ULTRA (USA)
GFAP, EP672Y, (05269784001)	Ventana, USA	ULTRA CC1, pH 8.45–8.65, 56 min, 25 °C	RTU *	16 min, 37 °C	OptiView DAB Detection Kit (Ventana Medical Systems Inc., Tucson, AZ, USA)	Ventana Benchmark ULTRA (USA)
*Sox10*, EP268 (383R-15)	Cell Marque, USA	Citrate buffer, pH 6.0, MW (1100 W), 20 min	1/100	60 min, 23 °C	DAKO REAL^TM^ EnVision Detection System Peroxidase/DAB+, Rabbit/Mouse (Dako, Denmark)	/

* RTU: ready to use. IHC: immunohistochemistry; MW: microwave oven.

**Table 3:** Signalment, clinical features, and tumor localization of the dogs included in the study.

No.	Breed	Age (Years)	Sex	Clinical Presentation	Location	Group
1	Cocker Spaniel	13	M	Central vestibular syndrome. Compulsive gait.	Left V. and VII. cranial nerve.	A
2	Labrador Retriever	6	M	Head tilt to the left.	Left V. nerve.	A
3	French Bulldog	6	M	NR	Lateral mass on the left *medulla oblongata* and *pons*.	A
4	Golden Retriever	10	M	NR	Right pontomesencephalic extra-axial neoplasia.	A
5	Mixed breed	7	M	Paralysis of the right VII., IX., and X. cranial nerves.	Right VII., IX., and X. cranial nerves.	A
6	Mixed breed	12	F	NR	Left trigeminal nerve.	A
7	Mixed breed	13	M	NR	Extradural mass of the right cervical spinal cord segment (C1–C6).	B
8	Yorkshire Terrier	12	F	NR	Intramedullary lesions at the level of C2 and C6.	B
9	Labrador Retriever	4	M	NR	Lardaceous extradural neoplasia C1–C2.	B
10	Rottweiler	9	F	NR	Tumor of the right C1–C2.	B
11	German Shepherd	4	M	NR	Medullary lesion of the cervical spine.	B
12	Beagle	8	M	NR	Intradural extramedullary neoplasia C4–C5.	B
13	Cane Corso	8	M	Bilateral flexor hyporeflexia and proprioceptive deficit of the right forelimb. Neck pain.	Neoplasia of the right C4 with medullary infiltration.	B
14	Mixed breed	8	M	Left hemiparesis.	Intradural extramedullary mass on the left C2–C3.	B
15	French Bulldog	8	F	NR	Neoplasia of C2 with compression of the spinal cord.	B
16	Golden Retriever	4	M	Progressive tetraparesis.	Epidural lesion C2–C4.	B
17	Staffordshire Terrier	9	M	NR	Neoplasia of the right C2 root with endocanalar extension and spinal cord compression.	B
18	Mixed breed	6	F	Progressive ataxia with severe proprioceptive deficits and cervical pain.	Intradural, extramedullary mass at the level of right C2.	B
19	Mixed breed	8	M	Lameness/paresis of the thoracic limbs (LMN and UMN type).	Below the spine at the level of C6–T1, extending upward through the foramina and infiltrating the epidural space.	C
20	Mixed breed	7.5	F	Atrophy of the right shoulder. Right pleurothotonus.	Extra- and intradural lesions at the level of C6–C7	C
21	English Setter	7	M	Postural deficit, hyporeflexia of the right forelimb.	Nerve roots involvement at the level of the cervicothoracic spine.	C
22	Mixed breed	8	M	NR	Nerve roots at the level of C7–T1	C
23	Dalmatian	11	M	Lameness of the left forelimb with muscular atrophy. Reduced forelimbs proprioception. Neck pain.	Nerve roots at the level of the C6–T1.	C
24	Yorkshire Terrier	7	M	NR	Intradural extramedullary lesion at the level of C7–T1.	C
25	Mixed breed	11	M	Chronic lameness and paresis of the left forelimb. EMG: denervation atrophy.	Nerve roots—T1.	C
26	Mixed breed	8	M	Neck pain and lameness of the right forelimb.	Intradural extramedullary neoplasia of the roots C6–C7.	C
27	German Shepherd	12	M	NR	Nerve root C8.	C
28	German Shepherd	12	M	Progressive left hemiparesis, progressing to recumbency.	Extra- and intravertebral neoplasm at the level of the left foramina C5–C6.	C
29	German Shepherd	6	M	NR	Left axillary region—the T2 root.	C
30	Maltese	6	F	NR	Involvement of the nerve roots C7–T1.	C
31	Mixed breed	12	M	Lameness of the right forelimb associated with hypomyotrophy.	Tumor of the nerve roots at the cervicothoracic spinal cord.	C
32	Mixed breed	6	F	Atrophy of the muscles of the shoulder and left forelimb.	Tumor of the nerve roots at the cervicothoracic spinal cord.	C
33	Czechoslovakian Wolfdog	11	M	NR	Nerve root C8.	C
34	Mixed breed	11	M	NR	Nerve root of the left C7.	C
35	Mixed breed	7	M	NR	Tumor of the nerve roots at the cervicothoracic spinal cord.	C
36	Labrador Retriever	7	M	NR	Endocanalar, extramedullary C6 lesion.	C
37	West Highland White Terrier	12	M	Progressive hemiparesis for 15 days.	Intradural extramedullary mass involving the nerve roots at the level of the cervicothoracic spinal cord.	C
38	Dogo Argentino	5	F	NR	Tumor of the right C6.	C
39	Mixed breed	11	F	Right forelimb lameness, decreased proprioception, and pain. Right Horner syndrome. Absence of panniculus reflex cranial to right T11.	Right axillary mass extending to the spinal cord by multiple nerve roots.	C
40	German Shepherd	10	F	Right forelimb lameness, flexor areflexia, and muscular atrophy.	Right C8 nerve.	C
41	German Shepherd	7	F	Left forelimb paresis and hyporeflexia.	Left C8 nerve root.	C
42	Bernese Mountain dog	7	M	Ataxia of the four limbs and neck pain.	NR	C
43	French Bulldog	10.5	F	NR	Neoplasia of the right C7 root.	C
44	German Shepherd	11	F	Acute paraparesis-paraplegia.	Tumor at the level of T8-T9 with involvement of the left nerve root.	D
45	Labrador Retriever	10	M	NR	Extradural mass at the level of L1–L2—lateralized on the left.	D
46	Labrador Retriever	3	M	NR	Lesion of the T9–T10.	D
47	Mixed breed	11	M	Right paraparesis, ataxia, and proprioceptive deficit.	Nerve root T13.	D
48	Mixed breed	10	M	NR	Neoplasia of the left root L3. Invasion of the spinal canal—intramedullary growth.	D
49	Fox Terrier	10	F	Vestibular syndrome, facial paralysis, bilateral progressive paraparesis (LMN type), paralysis of the urinary bladder.	Nerve roots at the level of L3–L5.	D, E
50	Mixed breed	13	F	NR	T13 and L5 nerve roots.	D, E
51	Mixed breed	8	M	Paraplegia (LMN type) and absence of deep pain perception.	Lumbosacral extra- and intradural lesion	E
52	Newfoundland dog	1.5	M	Lameness of the left hindlimb with impaired proprioception.	Tumor located ventral to the left transverse process of L7, adjacent to the nerve root L6. The tumor is encapsulated proximally and continues distally within the nerve.	E
53	Mixed breed	9	M	NR	Nerve roots involvement at the level of the lumbosacral spinal cord.	E
54	Mixed breed	4	M	Paraparesis with proprioceptive deficit, urinary and fecal incontinence.	Intradural, intramedullary mass L4–L7.	E
55	Cavalier King Charles Spaniel	12	M	NR	Lumbar paravertebral lesion on the left side.	E
56	Staffordshire Terrier	8	F	Right hindlimb paresis.	Extramedullary mass at the level of the L4–L5 nerve roots.	E
57	Mixed breed	6	M	NR	Right brachial plexus (C6–C7)	F
58	Beagle	8	M	NR	Brachial plexus.	F
59	German Wirehaired Pointer	8	M	Left forelimb paresis.Absence of spinal reflexes. Pain on palpation of the axilla.	Extramedullary centripetal lesion at the root of the left radial nerve.	F
60	Shih-Tzu	4	F	Pulmonary and brain metastases.	Brachial plexus.	F
61	Labrador Retriever	6	M	NR	Right brachial plexus.	F
62	Mixed breed	6	M	NR	Left brachial plexus.	F
63	English Setter	11	M	NR	Neoplasia of the left brachial plexus (C7–T1).	F
64	Boston Terrier	8	M	NR	Brachial plexus.	F
65	Mixed breed	9	M	NR	Brachial plexus.	F
66	German Shepherd	8	M	NR	Brachial plexus.	F
67	Mixed breed	11	M	NR	Right brachial plexus.	F
68	Mixed breed	11	M	Progressive lameness of left forelimb.	A mass in the left shoulder region—involving the brachial plexus and cervicothoracic spinal cord C4–T7.	F
69	Mixed breed	11	M	NR	Left brachial plexus.	F
70	Papillon	7	M	Progressive pain of the right forelimb (radicular syndrome) and neck pain.	Right brachial plexus tumor (C1–T2).	F
71	Mixed breed	8	F	Chronic paresis of the left forelimb.	Left brachial plexus.	F
72	Jack Russel Terrier	6.5	M	Chronic lameness of the right forelimb.	Neoplasia of the right brachial plexus extending to the C6–T1 nerve roots.	F
73	West Highland White Terrier	10	M	NR	Lumbosacral plexus.	G
74	Mixed breed	9	M	NR	Lumbar plexus (L6–L7).	G
75	Labrador Retriever	7	F	Lameness and muscle atrophy of the right pelvic limb	Right femoral nerve.	H
76	Labrador Retriever	5	M	NR	Left sciatic nerve.	H
77	German Shepherd	12	M	NR	Left radial nerve.	H
78	Bernese Mountain dog	12	M	NR	Brachial nerve.	H
79	Boxer	6	M	Chronic lameness and pain of the left forelimb.	Left ulnar nerve.	H

M: male; F: female; LMN: lower motor neuron; UMN: upper motor neuron; EMG: electromyography; NR: not reported. The letter in the last column is referred to the group: A: cranial nerve; B: cervical spinal cord segment; C: cervicothoracic spinal cord segment; D: thoracolumbar spinal cord segment; E: lumbosacral spinal cord segment; F: brachial plexus; G: lumbosacral plexus; H: appendicular nerve.

**Table 4:** Tumor types and results of immunohistochemical stainings for each examined case.

No.	Tumor Type	Histological Grade (If Malignant)	*Sox10* Expression	Claudin-1 Expression	GFAP Expression	CNPase Expression	Proliferation Index Ki-67 (%)
1	MNST—divergent	III	−	−	−	−	ND
2	MNST—conventional	III	+	+	−	−	ND
3	MNST—conventional	III	+	+	+	−	11.8
4	MNST—conventional	II	−	−	−	−	19.0
5	MNST—conventional	II	−	++	−	−	9.7
6	MNST—conventional	II	+	++	−	−	11.0
7	MNST—conventional	II	−	−	−	−	ND
8	MNST—conventional	II	+	−	−	−	15.8
9	MNST—conventional	III	−	−	−	−	27.7
10	Schwannoma—classic	NA	+++	−	++	+	1.5
11	MNST—conventional	II	+	+	−	−	9.1
12	Nerve sheath myxoma	NA	++	++	++	−	3.2
13	MNST—conventional	III	−	+	−	−	39.2
14	MNST—conventional	II	+	−	−	−	5.6
15	MNST—conventional	I	++	++	−	−	20.1
16	MNST—conventional	III	−	−	−	−	43.6
17	Neurofibroma	NA	++	+++	++	−	10.0
18	Neurofibroma	NA	++	++	+	−	6.4
19	MNST—conventional	III	−	−	−	−	ND
20	MNST—conventional	II	++	++	+	−	ND
21	MNST—conventional	III	(++)	(++)	(++)	−	ND
22	MNST—conventional	I	++	++	++	−	ND
23	Hybrid BNST— perineurioma/neurofibroma	NA	ND	++	ND	−	ND
24	MNST—conventional	I	++	++	(+)	−	ND
25	MNST—conventional	I	−	−	−	−	ND
26	MNST—conventional	II	+++	+	−	−	ND
27	MNST—conventional	I	+	+++	+	−	ND
28	MNST—conventional	III	+	+	−	−	ND
29	Hybrid BNST— perineurioma/schwannoma	NA	++	++	+	−	ND
30	MNST—conventional	II	++	++	+	−	23.2
31	Neurofibroma	NA	++	+++	++	−	11.0
32	MNST—conventional	I	++	++	++	−	10.5
33	MNST—perineural	II	−	+++	−	−	35.3
34	MNST—conventional	I	++	++	+	−	14.2
35	MNST—conventional	II	++	++	+	−	45.3
36	MNST—conventional	II	+	+	+	−	23.5
37	MNST—conventional	I	++	++	−	−	16.4
38	Nerve sheath myxoma	NA	++	++	+	−	8.2
39	MNST—divergent	III	++	++	+	−	71.4
40	Neurofibroma—plexiform	NA	++	++	++	−	0.9
41	MNST—conventional	III	++	+++	−	−	48.7
42	Neurofibroma	NA	++	+	++	−	2.0
43	MNST—conventional	II	+	+	−	−	32.1
44	MNST—divergent	III	−	−	−	−	ND
45	MNST—conventional	II	−	+	−	−	ND
46	Neurofibroma	NA	−	++	+	−	0.8
47	MNST—conventional	II	+	−	−	−	11.3
48	MNST—conventional	II	+	++	+	−	20.7
49	MNST—conventional	I	+	++	−	−	ND
50	MNST—conventional	II	−	++	−	−	7.8
51	MNST—conventional	II	+	+	−	−	ND
52	Nerve sheath myxoma	NA	+	+	+	−	ND
53	MNST—conventional	III	−	+	−	−	16.2
54	MNST—conventional	I	+	(+)	(+)	−	2.4
55	MNST—conventional	III	+	+	+	−	13.0
56	MNST—conventional	III	−	−	−	−	19.6
57	MNST—conventional	II	−	−	−	−	ND
58	MNST—perineural	II	−	+++	−	−	ND
59	MNST—conventional	II	+	+	+	−	ND
60	MNST—conventional	III	−	−	−	−	ND
61	MNST—conventional	I	++	++	+	−	9.1
62	MNST—conventional	III	+	+	−	−	28.5
63	MNST—perineural	III	−	+++	−	−	20.1
64	MNST—conventional	III	++	+	−	−	21.0
65	MNST—perineural	III	−	+++	−	−	64.3
66	MNST—divergent	III	−	++	−	−	22.1
67	MNST—conventional	I	+	++	−	−	29.7
68	MNST—conventional	II	++	++	−	−	30.2
69	MNST—conventional	III	+	++	−	−	46.4
70	MNST—divergent	III	++	++	++	−	31.7
71	MNST—conventional	II	+	+	+	−	47.0
72	MNST - conventional	II	+	+++	++	−	29.3
73	MNST—conventional	II	−	−	−	−	27.4
74	MNST—conventional	II	++	+	+	−	30.1
75	MNST—conventional	I	++	−	−	−	ND
76	MNST—conventional	I	+	+	+	−	14.4
77	MNST—conventional	I	+	−	−	−	15.7
78	MNST—divergent	III	++	−	−	−	11.2
79	MNST—epithelioid	II	+++	−	−	−	20.4

MNST: malignant nerve sheath tumor; BNST: benign nerve sheath tumor; −: negative reaction; +: weak positive reaction; ++: moderate positive reaction; +++: strong positive reaction; ND: no data; NA: not applicable. The result in brackets indicates that the reaction may be limited to the nerve residues.

**Table 5:** Results of immunohistochemical staining for *Sox10*, claudin-1, GFAP, and CNPase in different subtypes and variants of canine NSTs.

Tumor Type	*Sox10* Expression	Claudin-1 Expression	GFAP Expression	CNPase Expression
**BNST**				
Neurofibroma *	−/++	+/++/+++	+/++	−
Schwannoma *	+++	−	+/++	−/+
Perineurioma *	−	++/+++	−	−
Nerve sheath myxoma	+/++	+/++	+/++	−
**MNST**				
Conventional	−/+/++/++	−/+/++/+++	−/+/++	−
Divergent	−/++	−/++	−/+/++	−
Perineural	−	+++	−	−
Epithelioid	+++	−	−	−

BNST: benign nerve sheath tumor; MNST: malignant nerve sheath tumor; −: negative reaction; +: weak positive reaction; ++: moderate positive reaction; +++: strong positive reaction. * Each component of hybrid nerve sheath tumors is considered separately in the table.

**Table 6:** Results of immunohistochemical analysis for *Sox10*, Claudin-1, GFAP, CNPase, and proliferation index Ki-67 in each subtype and variant of NSTs.

Tumor Type	*N*	*Sox10* Expression, *n* (%)	Claudin-1 Expression, *n* (%)	GFAP Expression, *n* (%)	CNPase Expression, *n* (%)	Proliferation Index Ki-67 (%) Range **** (Mean, SD)
**NST**	79	54/77 (68.4) **	59/79 (74.7)	33/78 (43.0) *	1/79 (0.1)	0.8–71.4 (21.2 ± 15.6)
**BNST**	12	10/11 (90.9) *	11/12 (91.7)	11/11 (100) *	1/12 (0.8)	0.8–11.0 (4.9 ± 4.1)
Neurofibroma ***	7	5/6 (83.3) *	7/7 (100)	6/6 (100) *	0/7 (0)	0.8–11.0 (5.1 ± 4.6)
Schwannoma ***	2	2/2 (100)	0/2 (0)	2/2 (100)	1/2 (50.0)	1.5
Perineurioma ***	2	0/1 (0) *	2/2 (100)	0/1 (0) *	0/2 (0)	ND
Nerve sheath myxoma	3	3/3 (100)	3/3 (100)	3/3 (100)	0/3 (0)	3.2–8.2 (5.7 ± 3.5)
**MNST**	67	44/66 (66.7) *	48/67 (71.6)	22/67 (32.8)	0/67 (0)	2.4–71.4 (24.4 ± 15.1)
Conventional	56	40/55 (72.7) *	41/56 (73.2)	20/56 (35.7)	0/56 (0)	2.4–48.7 (22.3 ± 12.6)
*Grade I*	15	13/14 (91.7) *	12/15 (76.9)	8/15 (46.2)	0/15 (0)	2.4–29.7 (14.7 ± 7.6)
*Grade II*	25	18/25 (72.0)	18/25 (72.0)	9/25 (36.0)	0/25 (0)	5.6–47.0 (22.1 ± 12.3)
*Grade III*	16	9/16 (56.3)	11/16 (68.8)	3/16 (18.8)	0/16 (0)	11.8–48.7 (28.7 ± 13.7)
Divergent	6	3/6 (50.0)	3/6 (50.0)	2/6 (33.3)	0/6 (0)	11.2–71.4 (34.1 ± 26.2)
*Grade III*	6	3/6 (50.0)	3/6 (50.0)	2/6 (33.3)	0/6 (0)	11.2–71.4 (34.1 ± 26.2)
Perineural	4	0/4 (0)	4/4 (100)	0/4 (0)	0/4 (0)	20.1–64.3 (39.9 ± 22.5)
*Grade II*	2	0/2 (0)	2/2 (100)	0/2 (0)	0/2 (0)	35.3
*Grade III*	2	0/2 (0)	2/2 (100)	0/2 (0)	0/2 (0)	20.1–64.3 (45.2 ± 31.3)
Epithelioid	1	1 (100)	0/1 (0)	0/1 (0)	0/1 (0)	20.4
*Grade II*	1	1 (100)	0/1 (0)	0/1 (0)	0/1 (0)	20.4

N: number of all samples that match the diagnosis/variant/grade. *n*: number of samples expressing the IHC marker relative to the number of samples evaluated. NST: nerve sheath tumor. BNST: benign nerve sheath tumor. MNST: malignant nerve sheath tumor. * Staining for one sample was unreliable. ** Staining for two samples was unreliable. *** Each component of hybrid nerve sheath tumors is considered separately in the table. **** The value includes samples for which evaluation of the Ki-67 proliferation index was possible. SD: standard deviation. ND: no data.

## Data Availability

Data is contained within the article or Supplementary Material.

## Supplementary Materials

The following supporting information can be downloaded at: https://www.mdpi.com/article/10.3390/vetsci9050204/s1, Table S1: Evaluation of the tissue and cellular criteria of nerve sheath tumors.
