# Supplementary material for: Histopathological and Immunohistochemical Evaluation of Canine Nerve Sheath Tumors and Proposal for an Updated Classification
Source: Vet Sci. 2022 Apr 22;9(5):204. doi: 10.3390/vetsci9050204 (PMC9144584; doi:10.3390/vetsci9050204)
Supplement: Supplementary file 1 [file vetsci-09-00204-s001.zip › vetsci-1654089-supplementary.pdf]

Table S1: Evaluation of the tissue and cellular criteria of nerve sheath tumors.

**TISSUE CRITERIA**	
Circumscription	• Well circumscribed<br>• Ill-defined
Encapsulation	• Encapsulated<br>• Capsule invaded<br>• Incomplete fibrous pseudocapsule<br>• Unencapsulated
Shape	• Nodular / multilobular / cystic / other:
Cellularity	• Low<br>• Moderate<br>• High
Growth pattern;<br>The proportion of each growth pattern ((0) absent, (1) <25 %, (2) 25 – 50 %, (3) 50 – 75 %, (4) > 75 %)	Overall microscopical appearance:<br>- Heterogeneous<br>- Uniform<br><br>• Antoni type A (interlacing bundles, storiform, concentric)<br>• Antoni type B (loose textured)<br><br>Sheets / cords / meshwork of reticular fibers / rossettelike formations / sweeping fascicles / whorls / storiform pattern /plexiform pattern / other:
Stroma	Amount:<br>• Small<br>• Moderate<br>• Large<br>Type of stroma: collagenous / myxoid / fine /coarse / fibrovascular / fibrous / other:
Necrosis	• Absent<br>• Present, ≤ 50 %<br>• Present, > 50 %
Hemorrhages	• Absent<br>• Present
Vascular invasion	• Absent<br>• Blood vessel invasion<br>• Lymphatic vessel invasion<br>• Blood and lymphatic vessel invasion
Herniation into vessels	• Absent<br>• Present
Inflammatory infiltrates	• Absent<br>• Present<br>- Type: ________________________<br>- The extent of inflammation<br>- Location (perivascular, within the capsule, …):
Hyalinization	• Absent<br>• Present
Osteoid components	• Absent<br>• Present

Cartilaginous components	• Absent<br>• Present
**CELLULAR CRITERIA**	
Cellular morphology	Spindle / oval / polygonal / fusiform / onion-bulb-like formations / signet ring-like / epithelioid / other:
Anisocytosis	• Mild<br>• Moderate<br>• Strong
Anisokaryosis	• Mild<br>• Moderate<br>• Strong
Nuclear characteristics	Nuclear pleomorphism:<br>• Absent<br>• Present:<br>- Mild<br>- Moderate<br>- Strong<br>Nuclear shape: oval / round / elongated / bean-shaped / vesicular / hyperchromatic / other:
Nucleoli	• Not evident<br>• Prominent<br>- Number of nucleoli – up to _______<br>- Shape of nucleoli
Cytoplasm	Amount:<br>• Small<br>• Moderate<br>• Large<br>Color:<br>Character:<br>• *Homogenous*<br>• *Granular*<br>• *Containing pigment*<br>• *Other:*
Cell borders	• Distinct<br>• Indistinct
Mitoses<br>((1) 0-9/10 HPF, (2) 10-19/10 HPF, (3) >19/10 HPF)	1/ 2/ 3/ 4/ 5/<br>6/ 7/ 8/ 9/ 10/<br>MI = ______/10 HPF<br>Max. number of mitoses per HPF:<br>Presence of atypical mitotic figures:
Multinucleated cells	• Absent<br>• Present → Up to ________ nuclei
